# Platelet-rich plasma as a potential prophylactic measure against frozen shoulder in an in vivo shoulder contracture model

**DOI:** 10.1007/s00402-020-03617-x

**Published:** 2020-10-19

**Authors:** Oscar Feusi, Agnieszka Karol, Thea Fleischmann, Brigitte von Rechenberg, Samy Bouaicha, Clément M. L. Werner, Thorsten Jentzsch

**Affiliations:** 1 Department of Trauma, University Hospital Zurich, University of Zurich, Zurich, Switzerland; 2 Musculoskeletal Research Unit (MSRU) Center for Applied Biotechnology and Molecular Medicine (CABMM), University of Zurich, Zurich, Switzerland; 3 Division of Surgical Research, Centre for Clinical Research, University Hospital Zurich, University of Zurich, Zurich, Switzerland; 4 Department of Orthopaedics, Balgrist University Hospital, University of Zurich, Zurich, Switzerland

**Keywords:** Frozen shoulder, Adhesive capsulitis, Platelet-rich plasma, Shoulder, Injection, Infiltration

## Abstract

**Introduction:**

Frozen shoulder (adhesive capsulitis) is a common painful and functionally-limiting disease affecting around 2% of the population. So far, therapeutic options are limited and often unsatisfactory. Platelet-rich plasma (PRP) has been used as a treatment option in other orthopedic diseases since it contains growth factors that stimulate tissue repair. So far, the effect of PRP on frozen shoulder lacks evidence. We hypothesized that PRP may be valuable in the prophylaxis and treatment of secondary frozen shoulder due to capsular remodeling.

**Materials and methods:**

An experimental study of an in vivo frozen shoulder model was conducted. Twenty Sprague–Dawley rats underwent surgery in which the body of the scapula was connected to the humerus with a high-strength suture. Two groups of 8 weeks survival time were allocated; a treatment group with one intraoperative injection of PRP into the glenohumeral joint (*n* = 10) and a control group without PRP (*n* = 10). The primary outcome was the structural change in the posterior synovial membrane of the posterior and inferior part of the glenohumeral joint using a semi-quantitative grading from 0 (lowest) to 3 (highest).

**Results:**

The posterior synovial membrane structural changes were significantly lower in the PRP group (median = 1 [interquartile range (IQR) = 0–1]) compared to controls (median = 2 [IQR = 1–3]) (*p* = 0.028). There were no differences for the remaining synovial membrane changes and fibrous capsule responses between groups.

**Conclusions:**

In this in vivo shoulder contracture model, PRP injections seem to reduce the histological severity grade of some parts (i.e., posterior synovial membrane changes) of the secondary frozen shoulder without causing any side effects. It may be considered to investigate this effect further in future studies as a potential prophylaxis of secondary frozen shoulder (e.g., in operated or immobilized shoulders) or as a treatment option for patients with frozen shoulder in the early stage.

## Introduction

Frozen shoulder (also named adhesive capsulitis) is described as a slow-onset pain close to the insertion of the deltoid leading to sleep problems and painful restriction of elevation as well as external rotation without relevant changes on a conventional radiograph [1, 2]. It is a common disease affecting around 2% of the population [3] with a cumulative incidence of 2.4 per 1000 persons/year [4] and a peak incidence between 40–60 years [5]. Although it often resolves after 2 years, up to 40% of patients may not fully recover. Primary frozen shoulder is mostly idiopathic. Secondary frozen shoulder is often accompanied with a poorer prognosis and can be caused traumatically by fractures and soft-tissue injuries (e.g., rotator cuff tears and surgeries) or non-traumatically by impingement, calcific tendonitis, arthritis, and immobilization, which may cause inflammation [6] and contraction [7] of the glenohumeral joint [5, 8, 9]. Bunker et al. [10] concluded that various trigger factors, like minor traumas or injuries from surgery in the area around the shoulder, may evoke an inflammatory healing response in persons who are predisposed to contractures leading to pathological remodeling. However, at this point of time the basic pathogenesis of frozen shoulder essentially remains unknown.

Clinically, three stages can be observed: “freezing”, which includes night pain, active and passive stiffness, “frozen”, which shows reduced pain, and “thawing” which manifests less stiffness [5]. Most commonly, restrictions of external rotation are found due to capsular tightening [11, 12]. Histologically, inflammation and consecutive fibrosis with excessive type III collagen [10] and angiogenesis of the capsule and coraco-humeral ligament [13] can be seen. The latter is accompanied by a glassy appearance and pain in the beginning and fibrosis and stiffness later on [14]. The average duration of the disease is around 30 months, but it can also extend to more than 3 years [8, 15].

Unfortunately, the effects of the several treatment options, such as non-steroidal anti-inflammatory drugs, physiotherapy [16], steroid therapy [17], distension arthrography [18], manipulation under anesthesia [19], arthroscopic capsular release [20], and open surgical release [5] are often short-lived and are still controversially discussed in the literature. Since none of these treatment options have been clearly shown to be superior, further studies are needed in search for an effective prevention and treatment [5, 19, 21].

Platelet-rich plasma (PRP) is a bioactive part of whole blood and includes a variety of growth factors, some of which are assumed to influence remodeling, thus initiating and stimulating tissue repair [22–24]. It likely prevents an overwhelming inflammatory reaction by interacting with macrophages leading to an improvement of tissue healing and regeneration [22]. So far, the use of PRP has not been studied extensively in frozen shoulder cases and there are, to the best of our knowledge, only a few previous studies about this subject [25–27]. PRP might have a positive impact on the discussed disturbed composition of cytokines and growth factors in frozen shoulder.

In this study, we used an established in vivo contracture model in rats [28, 29] to compare the histological outcomes of a treatment group that received intraarticular PRP injections intraoperatively as a prophylactic measure and a control group without intraoperative PRP injections. We hypothesized that PRP may be valuable in prevention of frozen shoulder and in the early stages of the condition due to its presumed anti-inflammatory effects and the benefits described in literature regarding capsular remodeling.

## Materials and methods

### Ethics statement

Animal housing and all procedures and protocols were approved by the Cantonal Veterinary Office, Zurich, Switzerland (license number 279/2014). They were also in accordance with Swiss Animal Protection Law and were also conform to European Directive 2010/63/EU of the European Parliament and of the Council of 22 September 2010 on the Protection of Animals Used for Scientific Purposes and to the Guide for the Care and Use of Laboratory Animals.

### Study design

Twenty-three male Sprague–Dawley rats were obtained from a commercial supplier at the age of 12 weeks and were housed in groups of 2–3 animals under standardized conditions throughout the study. After an acclimatization period of 1 week, 20 rats were assigned randomly to 2 groups and underwent surgery to induce secondary frozen shoulder syndrome. Rats in the treatment group received one intraoperative injection of PRP into the glenohumeral joint (*n* = 10), rats in the control group had surgery only without injection of PRP (*n* = 10). Three animals were used for PRP extraction. The animals were sacrificed 8 weeks after the surgery.

### Anesthesia and preparation

Anesthesia was induced in a sealed chamber using a mixture of 5% isoflurane and oxygen at a flow rate of approximately 600 ml/minute (ml/min) and was then maintained via a nose mask with 2–3% isoflurane and oxygen at a flow rate of 600 ml/min. After loss of protective reflexes an eye ointment was applied. Then the right shoulder was shaved and disinfected. Each animal received a subcutaneous injection of ketamine-hydrochloride (20 mg [mg]/kilogram [kg] body weight [BW]) for pain alleviation prior to surgery. After preparation, the animal was placed on a warming pad in lateral recumbency on the operating table. All animals were monitored continuously during the procedure and surgery was performed under aseptic conditions.

### Surgical technique

The previously described surgical technique by Ochiai et al. [29] and Kanno et al. [28] was used. After palpating the surface landmarks on the right shoulder, including the spine of the scapula and humerus, a posterior approach was carried out. The caudal border of the scapula was dissected with minimal elevation of the subscapularis and infraspinatus muscles without damaging the glenohumeral joint capsule. Subsequently, a 2-0 FiberWire (Arthrex, Naples, United states of America [USA]) was used to connect the body of the scapula with the humerus, where it was passed around and firmly tied. The wound was closed in two layers, using 5-0 Vicryl for the subcutis and 4-0 Maxon for intracutaneous suture.

### Platelet-rich plasma

The preparation and the injections of PRP were done similar to the instructions given by Lamplot et al. [30, 31]. Platelet-rich plasma from three donor rats was used. Induction and maintenance of anesthesia was conducted as described above. The anesthetized animal was placed in dorsal recumbency, the thorax opened and the animal was euthanized. An intracardiac puncture with a 22-gauge needle of the left ventricle was performed to draw around 8 milliliters [ml] of whole blood that was mixed with 0.25 ml of citrate phosphate buffer. Separation of platelets was achieved by centrifugation in conical tubes of 15 ml at 200 g for 20 min at + 20° Celsius before the supernatant was centrifuged in a new set of conical tubes at 440 g again. After removal of the supernatant the precipitated platelets were harvested and mixed with the supernatant to reach a concentration of 1.5 × 10^9^/ml in a Bürkner chamber. The treatment group received one intraoperative injection of 10 microliters [μl] of PRP (around 10^9^ platelets/ml) into the glenohumeral joint prior to wound closure.

### Postoperative procedures

Analgesia was provided by subcutaneous applications of buprenorphine (0.1 mg/kg BW) every 6 hours during the day and by giving buprenorphine orally over the drinking water during the night for up to 3 days following surgery. All rats were monitored and scored daily for weight loss, general appearance, activity, locomotion and wound healing for a period of 5 days after surgery and afterward twice a week until the end of the experiment. Although a decrease of abduction and rotation in the right extremity and shoulder was observed, locomotion was not significantly impaired and all animals exhibited normal behavior, i.e., grooming or social interaction. On the last day of the experiment, 8 weeks after surgery, the animals were sacrificed with carbon dioxide and the shoulders were harvested and fixed in formalin for histological staining.

### Histopathology

To prevent damaging the capsule, the shoulders were dissected from the muscles after X-ray control using the X-Ray System Model LX-60 Faxitron (Faxitron X-Ray Corporation, Tucson, USA). Two thirds of the medial scapula and the distal part of the humerus were removed so that they could be placed in cases for paraffin embedding. Later on, the samples were decalcified in 25% Ethylenediaminetetraacetic acid [EDTA]﻿, pH 7–7.4 for 2 months and after cutting them into two halves through the glenoid, they were put in EDTA for another 2 weeks. Afterwards, the samples were dehydrated through a series of ascending alcohol concentrations, waxed after defatting in xylene and cast into paraffin-blocks. The Leica RM2235 microtome (Leica Microsystems GmbH, Wetzlar, Germany) was used to cut the blocks into 2–4 µm paraffin slides. Finally, the slides were de-paraffinized and stained with haematoxylin and eosin (HE).

All 20 slides were evaluated in a double-blinded fashion by one investigator and one independent observer under light microscopy. Structural changes characterized by tissue response (hyperemia, edema) and synovial membrane remodeling (folding and flattening of the membrane, severity grade of the atrophy of synovial epithelium and severity grade of the fibrosis of the subsynovial tissue layer) were evaluated and classified as either weak, moderate or strong deviation from the physiological structure. The inflammatory response in the synovial membrane and in the fibrous capsule of the posterior and inferior part of the glenohumeral joint were additionally tabulated as the outcome variable. The scoring criteria (Table 1) were modified based on the following studies [10, 13, 14, 32–36] and determined according to the histopathological changes within the examined groups.

**Table 1:** Scoring criteria of histopathological changes in the glenohumeral joint (synovial membrane, fibrous capsule), modified based on the following studies [10, 13, 14, 32–36]

	Grading	Synovial membrane	Fibrous capsule
Structural changes			
None	0	None	None
Weak	1	Hyperemia Mild synovial epithelium atrophy Minimally flattened synovial folds (5–30%)	Hyperemia Angiogenesis (3–7 vessels/visual field (10 ×)) Mild capsular fibrosis (10–20%)
Moderate	2	Edema Hyperemia Moderate synovial epithelium atrophy Partly flattened synovial folds (40–60%) Mild-moderate subsynovium fibrosis (20–60%)	Edema Hyperemia Angiogenesis (8–12 vessels/visual field (10 ×)) Moderate capsular fibrosis (30–60%)
Strong	3	Edema Hyperemia Severe synovial epithelium atrophy Mostly flattened synovial folds (70–100%) Severe subsynovium fibrosis (70–100%)	Edema Hyperemia Angiogenesis (> 12 vessels per visual field (10 ×)) Severe capsular fibrosis (70–100%)
Inflammatory response			
None	0	None	None
Weak	1	Scattered/focal appearing inflammatory cells	Scattered/focal appearing inflammatory cells
Moderate	2	Several/multifocal appearing inflammatory cells	Several/multifocal appearing inflammatory cells
Strong	3	Large number of/multifocal confluent inflammatory cells	Large number of/multifocal confluent inflammatory cells

Setting: 5 × -, 10 × -, 20 × -, 40 ×-magnification, haematoxilin-eosin-staining, Leica Digital-Modul-R (DMR), conventional light microscopy

### Statistics

Statistical analysis was performed using Stata software (Version 13.1; StataCorp LLC, College Station, Texas, USA). Data are presented as medians and interquartile ranges (IQR) due to non-normal distribution. For better overview of the semi-quantitative categorical/continuous data, both, the chi-squared and Wilcoxon rank sum tests, are provided. For the chi-squared test, weak/modest (grades 0–2) changes/responses were grouped and compared to a strong (grade 3) response.

*p* values less than 0.05 were considered significant.

## Results

Throughout the in vivo phase, there were no clinically visible postsurgical complications. All animals ambulated in the same fashion, exhibiting some mild limping at the beginning of the follow-up phase due to an altered positioning anatomy, which disappeared in the course of the follow-up period. It was therefore surprising that three fractures of the humeral metaphysis in the control group were detected in the post mortal radiographic images, which could be described histopathologically as an “intra-vitam” process.

**Fig. 1:**
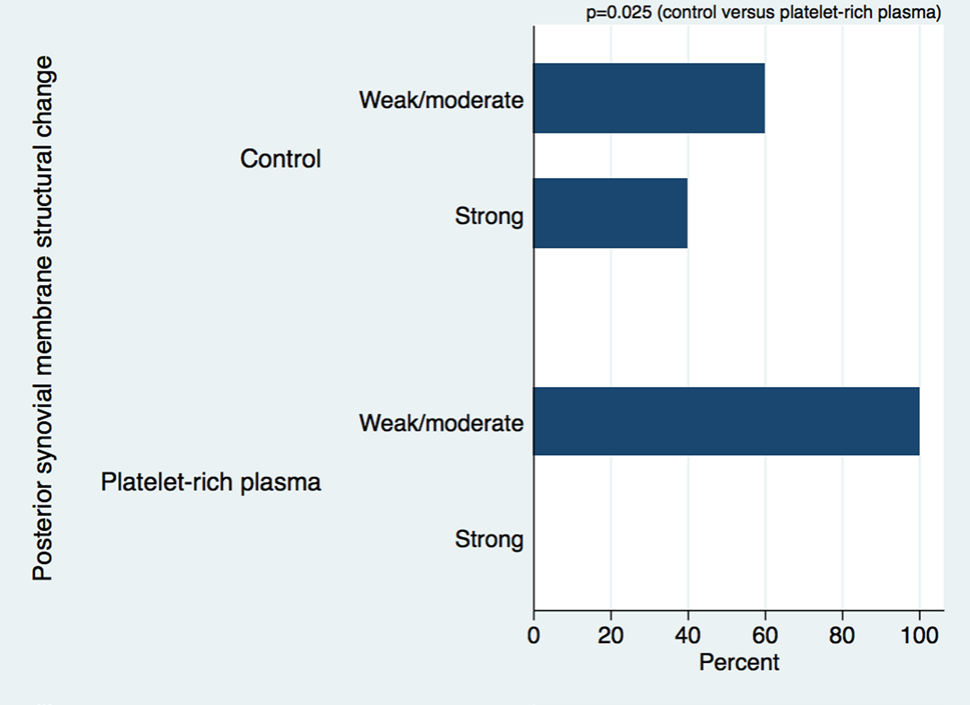
Semi-quantitative categorical data display of posterior synovial structural membrane changes in controls and treatment group in an in vivo shoulder contracture model (*n* = 20) (*p* = 0.025)

**Fig. 2:**
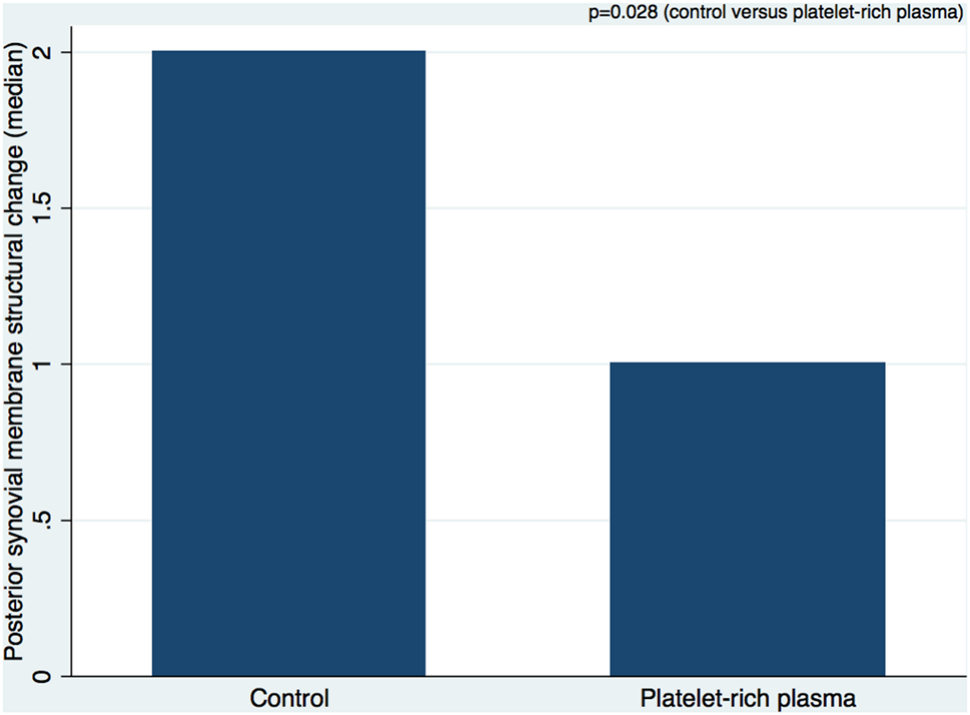
Semi-quantitative continuous data display of posterior synovial structural membrane changes in controls and treatment group in an in vivo shoulder contracture model (*n* = 20) (*p* = 0.028)

The structural changes in the posterior synovial membrane were significantly lower in animals that were treated with PRP compared to controls (chi-squared test: *p* = 0.025; Wilcoxon rank sum test: *p* = 0.028) (Figs. 1 and 2 and Tables 2 and 3). There were no significant differences for the remaining synovial membrane changes and fibrous capsule responses between the two groups. Further details of these results are given in the following paragraphs.

**Table 2:** Semi-quantitative categorical data display of synovial membrane changes and fibrous capsule responses in controls (*n* = 10) and treatment group (*n* = 10) in an in vivo shoulder contracture model (*n* = 20)

Variable	Group	Change/response				*p* value*
		Weak/moderate (0–2)			Strong (3)	
		0	1	2	3	
		*N* (%)	*N* (%)	*N* (%)	*N* (%)	
Posterior						
Synovial membrane						
Structural change	Control	1 (10)	3 (30)	2 (20)	4 (40)	0.025^†^
	PRP	4 (40)	4 (40)	2 (20)	0 (0)	
Inflammatory response	Control	8 (80)	2 (20)	0 (0)	0 (0)	na
	PRP	9 (90)	1 (10)	0 (0)	0 (0)	
Fibrous capsule						
Structural change	Control	2 (20)	7 (70)	1 (10)	0 (0)	na
	PRP	3 (30)	6 (60)	1 (10)	0 (0)	
Inflammatory response	Control	9 (90)	1 (10)	0 (0)	0 (0)	na
	PRP	10 (100)	0 (0)	0 (0)	0 (0)	
Inferior						
Synovial membrane						
Structural change	Control	2 (20)	6 (60)	1 (10)	1 (10)	0.305
	PRP	5 (50)	5 (50)	0 (0)	0 (0)	
Inflammatory response	Control	9 (90)	1 (10)	0 (0)	0 (0)	na
	PRP	10 (100)	0 (0)	0 (0)	0 (0)	
Fibrous capsule						
Structural change	Control	4 (40)	5 (50)	1 (10)	0 (0)	0.305
	PRP	3 (30)	7 (70)	0 (0)	0 (0)	
Inflammatory response	Control	9 (90)	1 (10)	0 (0)	0 (0)	0.305
	PRP	10 (100)	0 (0)	0 (0)	0 (0)	

*N* number, *%* percent, *PRP* platelet-rich plasma, *na* not applicable since all data were in the first (weak/moderate) category

*Chi-squared test comparing weak/moderate change/response (grade 0–2) versus strong (grade 3) response

^†^Significant

**Table 3:** Semi-quantitative continuous data display of synovial membrane changes and fibrous capsule responses in controls (*n* = 10) and treatment group (*n* = 10) in an in vivo shoulder contracture model (*n* = 20)

Variable	Group		*p* value*
	Control	PRP	
	Median (IQR)	Median (IQR)	
Posterior			
Synovial membrane			
Structural change	2 (1–3)	1 (0–1)	0.028^†^
Inflammatory response	0 (0–0)	0 (0–0)	0.542
Fibrous capsule			
Structural change	1 (1–1)	1 (0–1)	0.687
Inflammatory response	0 (0–0)	0 (0–0)	0.317
Inferior			
Synovial membrane			
Structural change	1 (1–1)	0 (0–0)	0.089
Inflammatory response	0 (0–0)	0 (0–0)	0.317
Fibrous capsule			
Structural change	1 (0–1)	1 (0–1)	0.895
Inflammatory response	0 (0–0)	0 (0–0)	0.317

*IQR* interquartile range, *PRP* platelet-rich plasma

*Wilcoxon rank sum test

^†^Significant

**Fig. 3:**
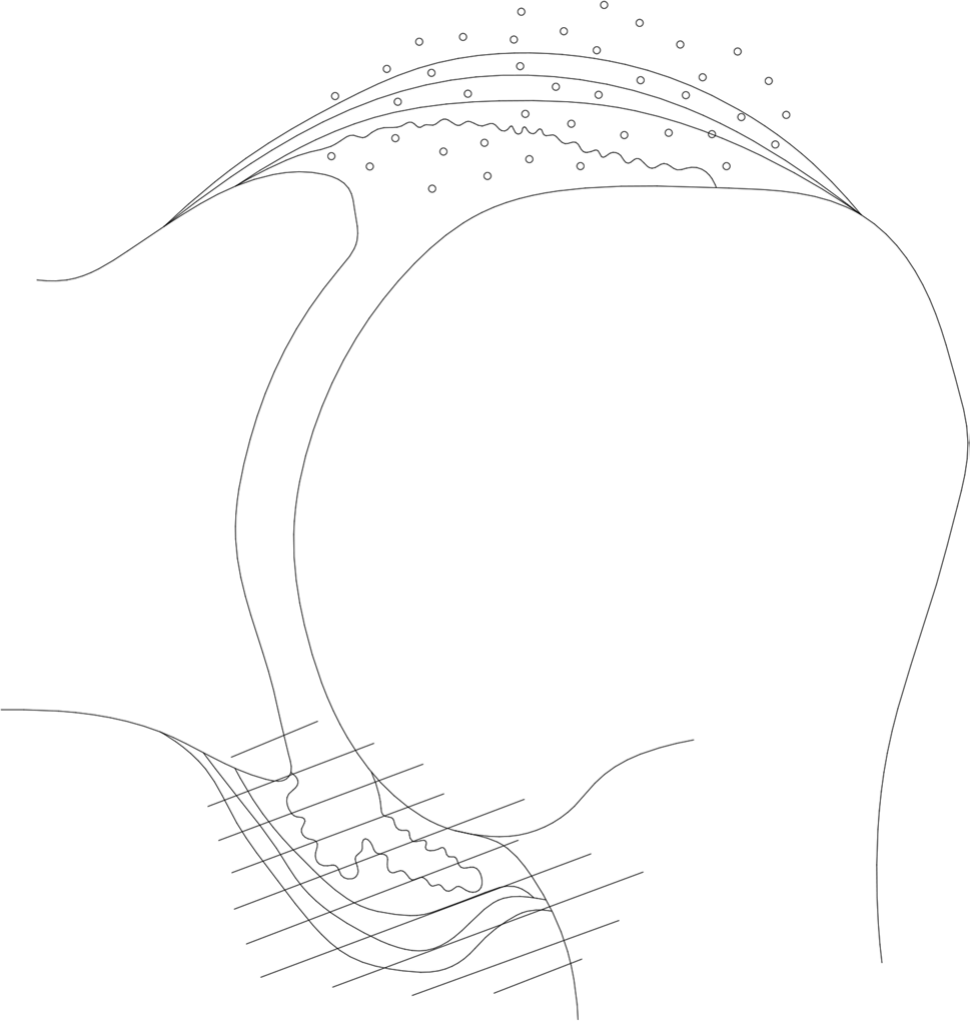
Schematic drawing of the areas under investigation. Spotted area represents the posterior part of the glenohumeral joint. Shaded area represents the inferior part of the glenohumeral joint

**Fig. 4:**
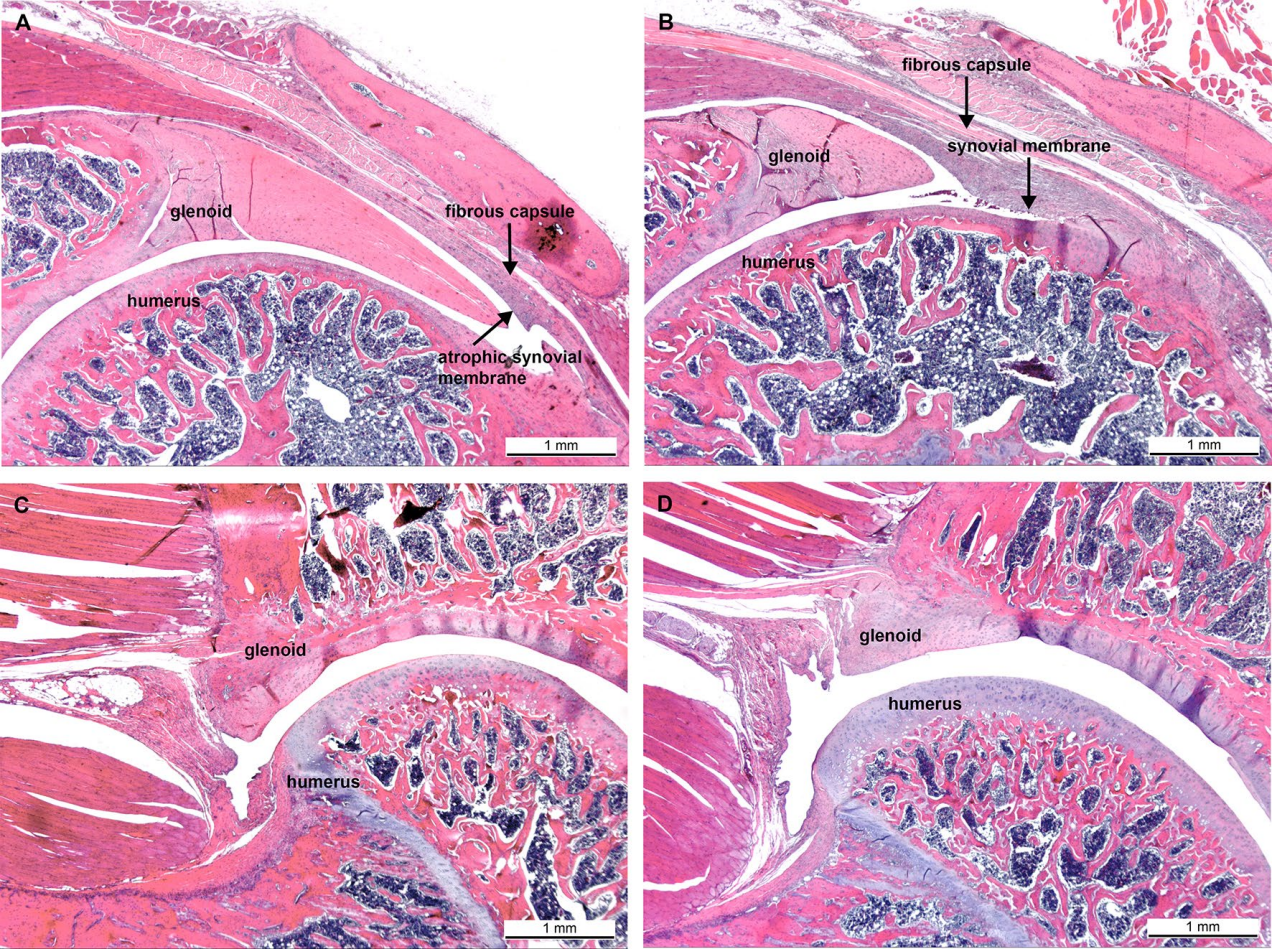
**a**–**b** Classification of the glenohumeral joint for the histological examination into the posterior part. **a** The control group shows an animal with strong structural changes in the synovial membrane and weak structural changes in the fibrous capsule without inflammatory response. **b** The platelet-rich plasma (PRP) group depicts an animal with weak structural changes in the synovial membrane and the fibrous capsule without an inflammatory response. Magnification × 125, HE staining. **c**–**d** Classification of the glenohumeral joint for the histological examination into the inferior part. **c** The control group represents an animal with weak structural changes in the synovial membrane and none structural changes in the fibrous capsule without inflammatory response. **d** The platelet-rich plasma (PRP) group presents an animal with none structural changes in the synovial membrane and weak structural changes in the fibrous capsule without inflammatory response. Magnification × 125, HE staining

**Fig. 5:**
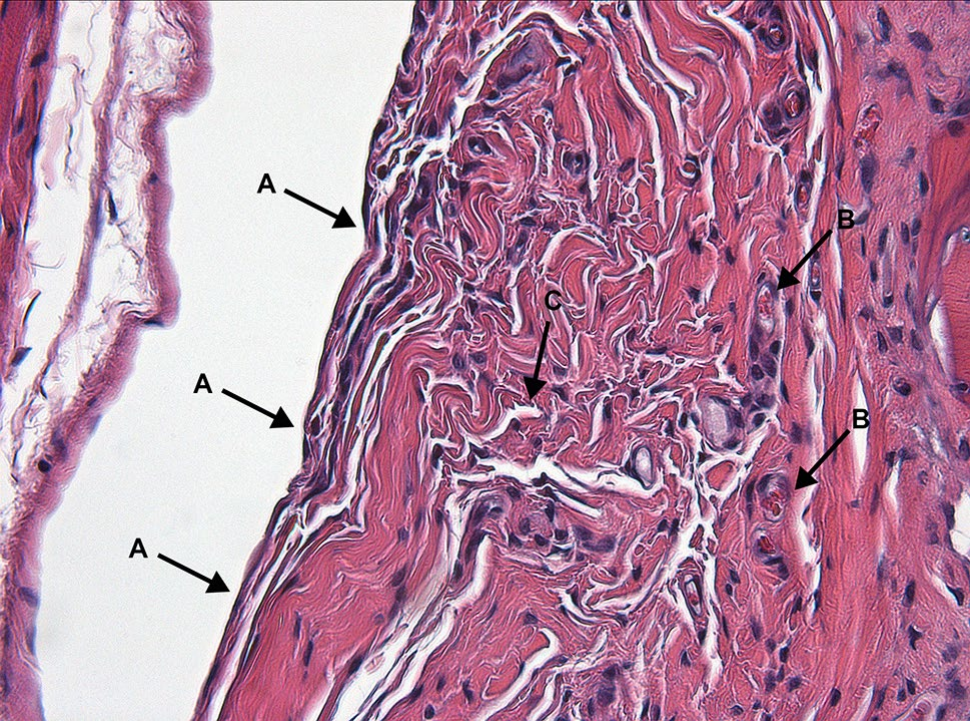
Posterior synovial membrane, control group; animal with strong structural changes in the synovial membrane. Arrows: **a** flattened synovial folds with loss/atrophy of synovial cells, **b** hyperemia, **c** edema. Magnification × 200, HE staining

**Fig. 6:**
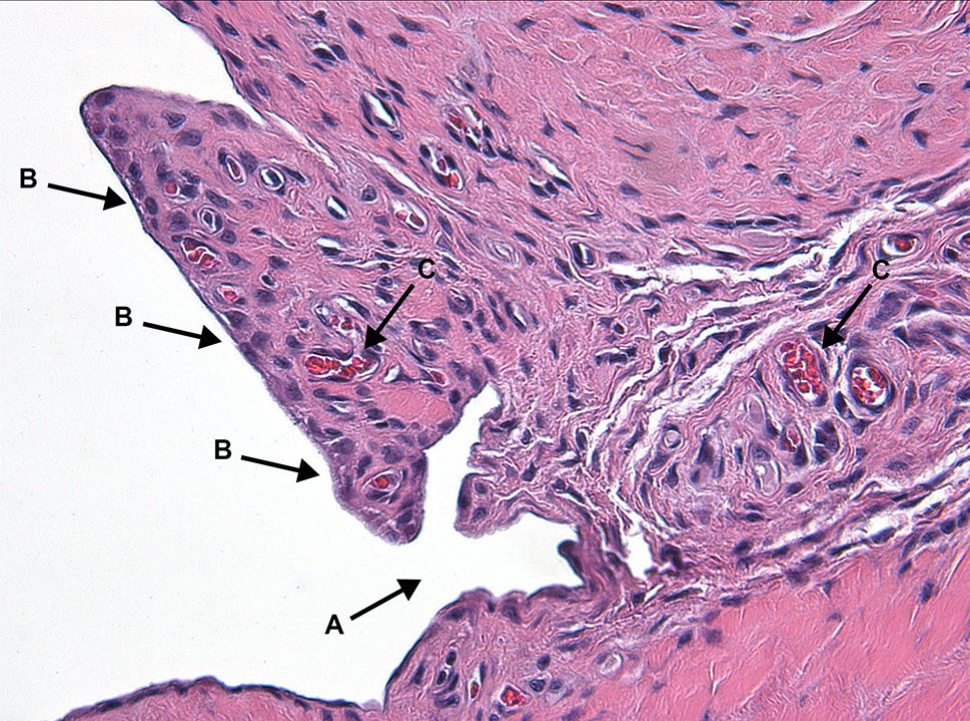
Posterior synovial membrane, PRP group; animal with weak structural changes in the synovial membrane. Arrows: **a** normal synovial folds, **b** normal synovial cells, **c** hyperemia. Magnification × 200, HE staining

**Fig. 7:**
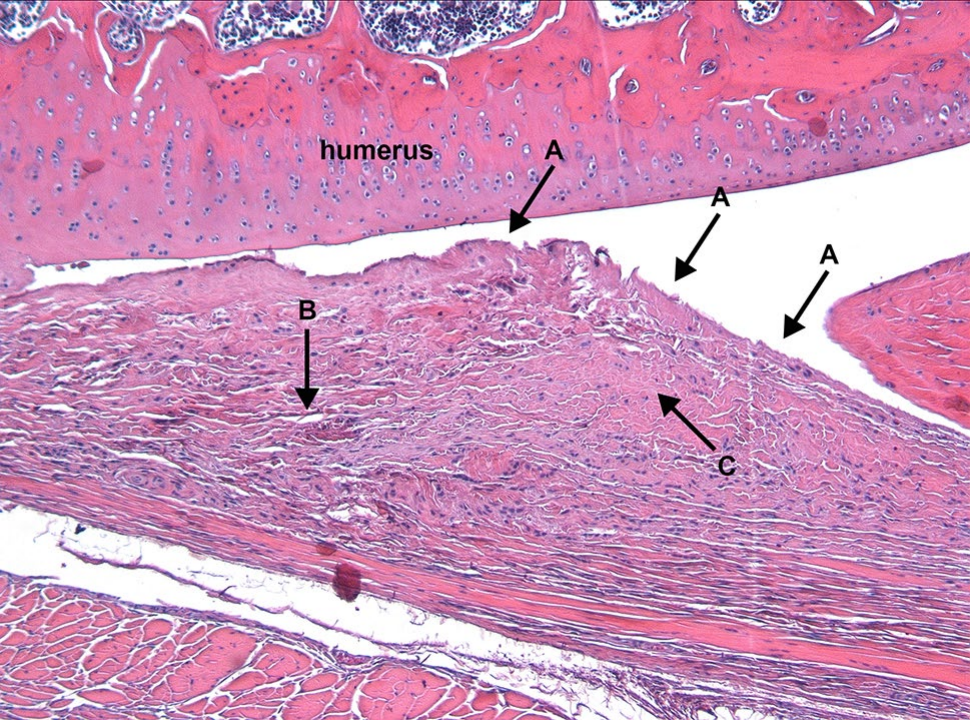
Posterior fibrous capsule, control group; animal with weak structural changes in the fibrous capsule and strong structural changes in the synovial membrane. Arrows: **a** flattened synovial folds with loss/atrophy of synovial cells, **b** edema, **c** severe fibrosis. Magnification × 50, HE staining

**Fig. 8:**
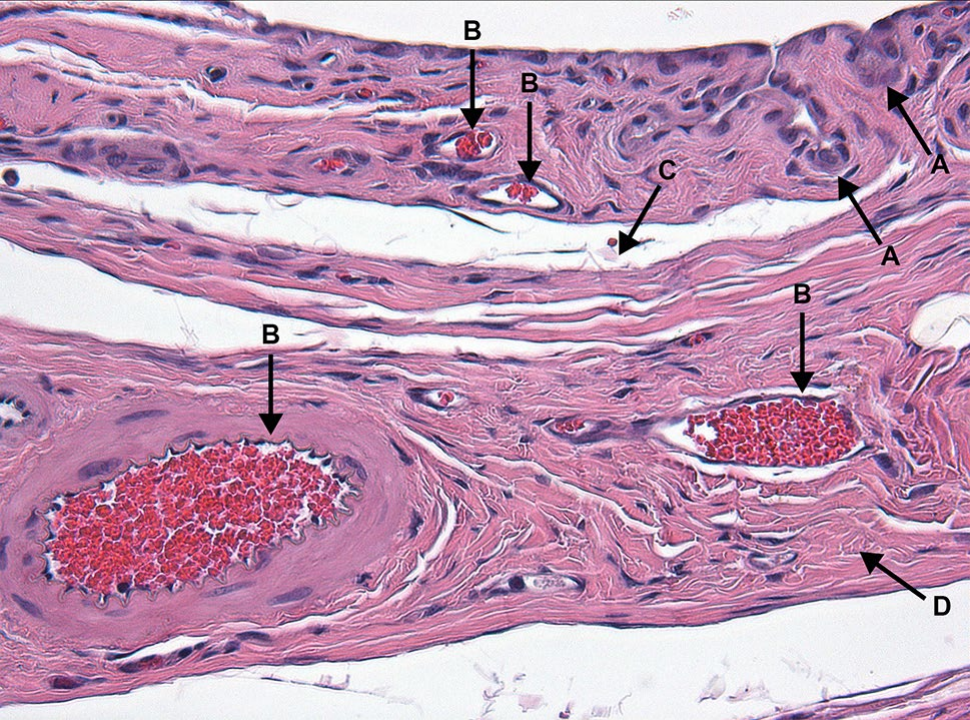
Posterior fibrous capsule, PRP group; animal with weak structural changes in the fibrous capsule and no structural changes in the synovial membrane. Arrows: **a** normal synovial folds with normal synovial cells, **b** capsular and subsynovial hyperemia, **c** edema, **d** mild fibrosis. Magnification × 200, HE staining

Figure 3 and 4 describe the classification of the glenohumeral joint into the posterior and the inferior part for the histological examination. Histological analysis of the affected shoulders revealed the following structural changes (Figs. 5, 6, 7 and 8): hyperemia, atrophy of the synovial epithelium, flattened synovial folds, edema in the synovial membrane, and fibrosis of the sub-synovium as well as hyperemia, angiogenesis, fibrosis, and edema in the fibrous capsule.

There was a weak to moderate inflammatory response present in two animals 2/10 (20%; number 2, 3) of the control group, but this finding was most likely due to fractures of the humeral metaphysis. In one animal (number 2 of the control group), a weak inflammatory response was found in the synovial membrane of both parts of the glenohumeral joint and in the fibrous capsule of the posterior part as well as a strong response in the inferior part of the glenohumeral joint. The other animal (number 3 of the control group) developed a weak inflammatory response in the synovial membrane of the posterior part of the joint.

One animal 1/10 (10%, number 8) of the PRP treatment group formed a weak inflammatory response located in the synovial membrane of the posterior part of the glenohumeral joint. No further inflammatory cells were present due to the secondary frozen shoulder.

The weak to strong structural changes in the synovial membrane of the posterior part of the glenohumeral joint were detected in nine animals 9/10 (90%; number 1, 2, 3, 5, 6, 7, 8, 9, 10) of the control group in comparison to six animals 6/10 (60%; number 3, 4, 5, 7, 9, 10) of the PRP treated group, where the structural changes were weak to moderate. Three animals 3/10 (30%; number 1, 5, 6) in the control group and four animals 4/10 (40%; number 3, 5, 9, 10) in the treatment group developed weak structural changes in this location. In both groups two animals 2/10 (20%, number 2, 3 in the control group and number 4, 7 in the treatment group) were found with moderate changes, respectively. No strong structural changes were detected in the synovial membrane of the posterior part of the glenohumeral joint in PRP treated group in comparison to the control group, where four animals 4/10 (40%; number 7, 8, 9, 10) resulted in strong structure changes in this region.

There was no significant difference of the structural changes in synovial membrane of the inferior part of the glenohumeral joint between the control and the PRP treatment group observed. Six animals 6/10 (60%; number 1, 3, 4, 5, 7, 9) of the control group and five animals 5/10 (50%; number 4, 6, 7, 9, 10) of the treatment group revealed some weak structural changes. In the control group one animal 1/10 (10%; number 8) showed moderate structural changes and in another one 1/10 (10%; number 10) strong structural changes were detected.

There was no major difference between the control and the treatment group regarding the changes in the fibrous capsule. One individual animal 1/10 (10%; number 2 in the control group and number 8 in the treatment group) from both control and PRP treatment groups revealed moderate structural changes in the posterior part of the glenohumeral joint. Seven animals 7/10 (70%; number 1, 4, 5, 6, 7, 9, 10) in the control group and six animals 6/10 (60%; number 1, 2, 3, 4, 5, 6) in the treatment group developed weak changes in this location.

In the inferior part one animal 1/10 (10%; number 2) from the control group showed strong structural changes in the fibrous capsule. This was interpreted as a result of the fracture of the bone with subsequent instability and increased mobility within the joint. 5/10 animals (50%; number 1, 4, 5, 8, 10) from the control group and 7/10 (70%; number 1, 2, 3, 4, 5, 6, 8) of the PRP treatment group showed some weak structural changes in the fibrous tissue of the inferior part of the glenohumeral joint.

## Discussion

According to the results of this study, PRP injection into the glenohumeral joint prevented strong structural changes in the posterior synovial membrane of rats in an in vivo shoulder contracture model. There were no clinical side effects observed due to PRP. To the knowledge of the authors, this is the first standardized experimental preclinical study conducted in rats proving a beneficial effect of PRP, probably by down regulating the inflammatory responses in this model of secondary frozen shoulder.

So far, there is very sparse evidence about the use of PRP in frozen shoulder and if so, has mainly been reported in clinical studies in humans. One case report by Aslani et al. [25] found beneficial results after intraarticular injection of PRP. This led to satisfactory improvements by 60%, 100%, and 70% in diurnal pain, night pain, and function, respectively. Furthermore, Kothari et al. [26] demonstrated a superior treatment effect of PRP in their clinical study in which 195 patients with frozen shoulder were treated randomized with a single injection of PRP or a corticosteroid or with ultrasonic therapy, each in combination with exercise therapy. After 12 weeks, the group receiving the PRP injection showed significant improvement over corticosteroid and ultrasonic therapy in active and passive range of motion, pain and function without suffering from any major side effects. Moreover, Lin et al. [27] conducted a randomized controlled trial with a 6-month follow-up, in which they revealed that PRP injections showed a better and longer positive effect than injections of a local anaesthetic in the treatment of 60 patients with frozen shoulder, each in combination with exercise therapy.

Our findings are in line with the descriptions in other studies [35, 36]. As the shoulders were harvested for histological analysis 8 weeks after surgery, the frozen shoulder was no longer in the early stage and there were no inflammatory infiltrates. At this point, the synovial membrane was no longer hyperplastic, but started to become atrophic and began to disappear. The tissue was often strongly vascularized and hyperemic.

As also seen in other shoulder diseases, the posterior capsule tends to become tight in frozen shoulder [37]. This was also observed in this experimental study in rats and could be one reason why significant results were found in the posterior capsule in this study. Although the pathogenesis of frozen shoulder is not completely understood yet [5], it likely involves an inflammatory [38] or fibrotic process [13]. It is also assumed that a cytokine modulated inflammatory process is followed by a reactive fibrosis [10, 14, 35], which is compatible with our findings and would correlate with the lengthened clinical process, i.e., the painful start and the following stiffness [5, 10, 14]. At the onset of the disease the synovium appears hypervascular and hyperplastic [35, 36]. Later, type III collagen accumulates in the subsynovium and the capsule and causes a fibrosis [35], which we have also seen in our specimens.

According to Hand et al. [14], inflammatory infiltrates can be found that mainly consist of mast cells, macrophages and T cells, whereas Bunker et al. [13, 39] found no immune cells apart from a few leukocytes and macrophages in the synovium and the area around the blood vessels, which were also observed in our samples. Additionally, the tissue was, as we have documented too, strongly vascularized. This has also been seen macroscopically and matches the presumption of an inflammatory process [14]. The cell population contained mostly proliferating fibroblasts [14] which produce type III collagen excessively [10]. Furthermore, Bunker et al. [10, 13, 39] reported the presence of myofibroblasts, which was also confirmed by Hettrich et al. [40] and could be the reason for the capsule contracture. In our study, myofibroblast identification was not performed and thus cannot be confirmed.

The fact that the frozen shoulder model was based on immobilization and soft-tissue injury due to the operation only is a limitation of our study. However, so far, no alternative (idiopathic) frozen shoulder models have been described to the best of our knowledge [28, 29, 36]. Since secondary frozen shoulder can be associated with a poorer prognosis than the primary form [5], it is possible that the effects of PRP as a treatment in the early stage of primary frozen shoulder would be stronger. Although rats have been described as the preferred species for this model, because the anatomy of their shoulder resembles the human one, the function of a rat shoulder might vary compared to a human shoulder [28]. Three humeral fractures were found in the control group, despite a clinically uneventful, monitored course. Histologically, the lesions were estimated to have occurred around 5 weeks before the sacrifice, in the earlier phase of the study. However, the exact point of time and reason remain uncertain. Therefore, radiographs may be recommended to exclude fractures. As expected, there were no side effects monitored during the treatment with PRP [22, 30]. Lastly, the sample size of this study was rather small, but due to the pilot nature sufficient to draw some preliminary conclusions.

Future studies may consider repeating a similar study focusing on the nature of the inflammatory response and the role of macrophages, cytokines, MMPs and growth factors such as tumor necrosis factor (TNF), transforming growth factor (TGF) and platelet-derived growth factor (PDGF). In the clinical setting, a randomized controlled trial may be set up with intraarticular PRP injections as prophylaxis against post-surgical capsular stiffness or shoulder contracture due to immobilization.

In conclusion, the rat frozen shoulder model can be considered as a somewhat feasible and reliable animal model to study pathology of the condition and its treatment. The PRP injection treatment did not cause any side effects and was safe. It seems to reduce the histological severity grade of the secondary frozen shoulder-induced alteration in the posterior synovial membrane. Although there were no significant differences for the remaining synovial membrane changes and fibrous capsule responses between groups, it could be contemplated to investigate this effect further in future studies as a potential prevention of post-operative stiffness and treatment option for patients with frozen shoulder in the early stage.
